# Officially Confirmed COVID-19 and Unreported COVID-19–Like Illness Death Counts: An Assessment of Reporting Discrepancy in Bangladesh

**DOI:** 10.4269/ajtmh.20-1205

**Published:** 2020-12-14

**Authors:** Mazbahul G. Ahamad, Fahian Tanin, Byomkesh Talukder, Monir U. Ahmed

**Affiliations:** 1 University of Nebraska–Lincoln, Lincoln, Nebraska;; 2 Independent Researcher, Sylhet, Bangladesh;; 3 Dahdaleh Institute for Global Health Research, York University, Toronto, Canada;; 4 Department of Economics, Shahjalal University of Science and Technology, Sylhet, Bangladesh

## Abstract

Reporting discrepancies between officially confirmed COVID-19 death counts and unreported COVID-19–like illness (CLI) death counts have been evident across the world, including Bangladesh. Publicly available data were used to explore the differences between confirmed COVID-19 death counts and deaths with possible COVID-19 symptoms between March 2, 2020 and August 22, 2020. Unreported CLI death counts totaled more than half of the confirmed COVID-19 death counts during the study period. However, the reporting authority did not consider CLI deaths, which might produce incomplete and unreliable COVID-19 data and respective mortality rates. All deaths with possible COVID-19 symptoms need to be included in provisional death counts to better estimate the COVID-19 mortality rate and to develop data-driven COVID-19 response strategies. An urgent initiative is needed to prepare a comprehensive guideline for reporting COVID-19 deaths.

## INTRODUCTION

Underreporting COVID-19 death statistics, by either mistake or purposefully changing death counts and reporting methodologies, is not uncommon across the world, with countries including China and Peru adjusting their death counts.^1–3^ The World Health Organization (WHO) has suggested including “death[s] resulting from a clinically compatible illness in a probable or confirmed COVID-19 case”^4^ in mortality statistics to support pandemic surveillance. However, evidence has emerged showing the Institute of Epidemiology Disease Control and Research (IEDCR), a reporting authority in Bangladesh, has only reported COVID-19 deaths and has not reported any COVID-19–like illness (CLI) deaths despite the suggested (e.g., WHO) guidelines.^4^

Between March 2 and August 22, 2020, a total of 3,907 COVID-19 deaths were officially recorded by the IEDCR.^5^ During the same period, a total of 2,156 people died with symptoms consistent with CLI, according to the Centre for Genocide Studies (CGS) in Bangladesh.^6^ This means that 2,156 officially unreported deaths may have been caused by COVID-19, which could have been reported as CLI deaths along with existing COVID-19 death surveillance and monitoring efforts. Mean unreported CLI death counts (86 deaths/week) were half of the officially confirmed COVID-19 death counts (156 deaths/week) until late August, which suggests a major discrepancy between comprehensive death data collection and reporting. COVID-19 death counts and mortality rates have a direct effect on research and policy implications as well as the selection of pandemic response strategies, and adequate documentation of both confirmed and unreported CLI death counts by the reporting authority has become essential for accurate reporting. We, therefore, compared officially confirmed COVID-19 and unreported CLI death counts to explore reporting discrepancies in the absence of a practically comprehensive COVID-19 death reporting guideline in Bangladesh.

## METHODS

We used publicly available data to explore trends and assess the differences between confirmed COVID-19 and unreported CLI death counts between March 2, 2020 and August 22, 2020 in Bangladesh. Officially confirmed daily COVID-19 death data were obtained from the Johns Hopkins Coronavirus Resource Center (accessed on August 23, 2020).^5^ Unreported CLI death counts were collected from the weekly reports of the CGS (accessed on August 25, 2020),^6^ which collects COVID-19–like death counts from daily newspapers, retraces each case, and removes duplication. We calculated the weekly confirmed COVID-19 death counts to be comparable to the weekly unreported CLI death counts. We also estimated the 2-week moving average of each death counts to show respective trends. We further considered the entire time line (March 2–August 22, 2020) and divided it into two different phases to present the trend and mean death counts of each phase. During the first phase (March 2–May 2, 2020), unconfirmed deaths were more numerous than confirmed deaths, whereas in the second phase (May 3–August 22, 2020), unconfirmed deaths totaled less than confirmed deaths. We used a *t*-test to compare mean confirmed and CLI death counts during the entire period and in both aforementioned phases. Statistical analyses were conducted using Stata (Version 16.1, StataCorp LLC, College Station, TX).^7^ Data used in the study will be made available publicly via Harvard Dataverse.^8^

## RESULTS

During the study period, the mean number of confirmed deaths was 156 (95% CI: 105.37–207.19) per week, whereas the mean number of unreported CLI deaths was approximately 86 (95% CI: 59.49–112.99) per week. Statistically significant mean differences existed between confirmed COVID-19 and unreported CLI death counts (*t =* 2.51; *P =* 0.02).

**Figure 1.**
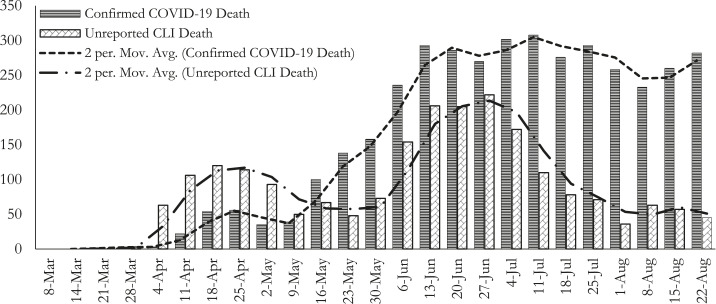
Weekly confirmed COVID-19 and unreported COVID-19–like illness (CLI) death counts, March 2–August 22, 2020, Bangladesh. The horizontal axis indicates the last date of the respective week.

Figure 1 presents the weekly counts of both confirmed COVID-19 and unreported CLI deaths along with the respective 2-week moving averages. Although both the reported confirmed and unreported CLI death counts show increasing trends over the study period in most of the weeks examined, unreported death counts have displayed a decreasing trend in recent weeks. For the week ending May 8, 2020, the confirmed COVID-19 death counts exceeded the unreported CLI death counts (Figure 1). Unreported CLI death counts were higher than confirmed COVID-19 death counts before this week (average: ∼55 versus 19), whereas it was lower in the following weeks (average: ∼104 versus ∼233) (Table 1). On average, the percentage of unreported CLI deaths was 49% of the total deaths (sum of confirmed and unreported deaths) from March 2 to May 2, 2020, decreasing to 31% of the total deaths from May 3 to August 22, 2020.

**Table 1:** Mean confirmed COVID-19 and unreported COVID-19–like illness death counts, March 2–August 22, 2020, Bangladesh

COVID-19 death category	March 2–May 2, 2020	May 3–August 22, 2020	t*	*P-*value†
Confirmed (mean, 95% CI‡)	19.4 (1.46–37.43)	233.3 (190.32–276.18)	−7.72	0.000
Unreported (mean, 95% CI)	55.4 (13.70–97.19)	103.6 (68.80–138.32)	−1.87	0.074

* Indicates *t*-statistic.

† Indicates probability value.

‡ Indicates confidence interval.

## DISCUSSION

The number of CLI deaths (2,156) that were unreported is high; more than half (55% of 3,907 confirmed deaths) of the total deceased who showed COVID-19–like symptoms were either not tested after death or not reported appropriately. On March 13, 2020, a report of the CGS raised public awareness of this issue,^9^ and subsequent correspondences^10^ also highlighted this discrepancy and its possible implications in an attempt to raise the awareness of the authorities responsible for COVID-19 mortality surveillance in Bangladesh. The reporting authority neither documented these deaths as resulting from CLI nor adjusted for potential seasonal influenza-like illness or other related deaths as per the relevant guidelines,^11,12^ which might have led to unclear COVID-19 data and mortality rates. This significant number of unreported CLI deaths may incorrectly suggest that the prevalence of COVID-19 in Bangladesh is very low; this, in turn, can be challenging for two different, yet interlinked perspectives as follows.

First, public health researchers and policy analysts cannot properly recognize the dynamics of COVID-19 without access to reliable data. Having incorrect or inadequate information will restrict their ability to accurately identify the current stage of the COVID-19 pandemic and predict peak case and death counts. Second, the death-reporting gap may present challenges when preparing COVID-19 response strategies, for example, in allocating coronavirus treatment hospitals and intensive care beds. Erroneous death counts can undermine ongoing response efforts and limit access to public health assistance, such as vaccines, coming from international organizations and development partners. One concern in particular should be observed in the context of its limitation: death counts from both officially confirmed COVID-19 and unofficial CLI might be different from the true or actual counts because of unaccounted out-of-hospital deaths and missed unreported CLI deaths in major newspapers.

## CONCLUSION

Incomplete COVID-19 death reporting constitutes a data gap and a challenge to understanding ongoing coronavirus pandemic dynamics, especially with regard to future waves. We recommend that all data collection and reporting authorities responsibly implement real-time tracking and reporting of CLI deaths as provisional together with confirmed COVID-19 deaths, complying with the WHO’s suggested surveillance approach toward reporting COVID-19 deaths,^3^ which may be similar to the reporting standard of the U.S. CDC.^13^ Having accurate data on both confirmed and provisional death statistics will enable public health researchers, policy analysts, and development partners to correctly estimate the COVID-19–related mortality rate and subsequently design data-driven pandemic preparedness efforts, early response strategies for forthcoming waves of the pandemic, and emergency actions with necessary assistance from both national and international public health organizations.
